# Effects of online social media on improving mothers’ behaviors towards preventing their children’s otitis media based on the PRECED model: a randomized educational intervention trial

**DOI:** 10.1186/s12887-023-04016-y

**Published:** 2023-05-05

**Authors:** Atefeh Moradi, Raheleh Soltani, Mohsen Shamsi, Rahmatallah Moradzadeh

**Affiliations:** 1 Student Research Committee, Department of Health Education, Faculty of Health, Arak University of Medical Sciences, Arak, Iran; 2 Department of Health Education, Faculty of Health, Arak University of Medical Sciences, Arak, Iran; 3 Department of Epidemiology, Faculty of Health, Arak University of Medical Sciences, Arak, Iran

**Keywords:** Otitis media, PRECEDE model, Mothers, Infants, Social media

## Abstract

**Background:**

Otitis media is one of the most common diseases in children, especially those under 2 years of age. This study aimed to investigate the effect of educational intervention based on the PRECEDE model on mothers’ preventive behaviors of middle ear infections in infants.

**Methods:**

This study was conducted as an educational randomized controlled trial on 88 mothers with infants referred to health centers in Arak, Iran. Sampling from September 2021 to February 2022 selected trough stratified random sampling who were assigned to two groups of experimental = 44 and control = 44. The data collection tool was a reliable and valid questionnaire that included demographic information, constructs of PRECEDE model regarding otitis media, and preventive behaviors. The experimental group received 4 training sessions (each session 60 min) through WhatsApp social network. Information was collected through an online questionnaire before and 3 months after the educational intervention from both groups. Data analysis was also performed with SPSS version 23.

**Results:**

Before the educational intervention there was no significant difference between the experimental and control groups in the otitis media preventive behaviors and structures of PRECEDE model (*p* > 0.05). After the educational intervention, in the experimental group the average score of knowledge from 0.49 to 0.81, attitude from 4.01 to 4.58, enabling factors from 0.72 to 0.85, reinforcing factors from 3.31 to 3.91 and behavior from 3.25 to 3.66 increased significantly (*p* < 0.001).

**Conclusions:**

PRECEDE-based education with controlling, monitoring and follow-up during the program was effective in promoting the preventive behaviors of otitis media. Therefore, due to the side effects of otitis media, especially in vulnerable periods such as childhood, it is recommended that trainings based on this model be carried out in other health care centers and clinics in order to maintain children health.

**Trial registration:**

This trial has been registered at the Iranian Registry of Clinical Trials, IRCT20210202050228N1. Prospectively registered at 2021-May-21, (2021/05/21) available at: URL: https://en.irct.ir/trial/54073.

## Introduction

Otitis media is one of the most common infections in children and the second most common disease after upper respiratory infection in children [1, 2]. Also, it is one of the most common health problems in developing and developed countries and one of the reasons why children visit a doctor and takes antibiotics [3]. Acute otitis media is more common in children aged 3–18 months [4]. According to studies, the prevalence of otitis media is different in different regions, and in general, about 80% of children, especially before school- age, experience otitis media at least once [5, 6]. The prevalence of otitis media in Brennan’s study in Australia at 5 to 7-year-old children was 22.5% [7]. A systematic review study reported the prevalence of otitis media in Iran as 9.1% [8] and in the study by Saki et al. [9], the prevalence of otitis media in children less than 6 years old was 31%. The latest available study (2020) reported the prevalence of acute otitis media in Iran as 51% in the right ear, 44% in the left ear, and 33% in both ears [10].

Otitis media has serious consequences and complications for children’s health and affects the quality of life of children and their families [11]. According to studies, its complications include the impact on the quality of life-related to education, learning disorders [12], disruption in daily communication and social interactions, constant release from the ear, and ear pain [13, 14]. One of the most common complications of this disease is hearing damage and loss, and one out of every 300 children less than 5-year-old suffers from hearing damage related to middle-ear infection [15].

Some of the risk factors in the occurrence of otitis media can be mentioned as lack of breastfeeding, family history, taking care of children outside of home and in crowded areas, and inhaling cigarette smoke [3]. Studies emphasize the role of knowledge, attitude and behavior of parents, especially mothers, for the prevention of otitis media [16–18]. In a study by Baghiani Moghadam, 4.5% of mothers with children less than 2-year-old had good knowledge about Otitis media [19]. In another similar study, only 30% of mothers performed optimally in the field of otitis media prevention behaviors, and this study emphasized the need for health care providers to pay more attention to this issue [20].

Studies also, emphasize the necessity of educational intervention in order to improve the knowledge, attitude and behavior of mothers regarding otitis media [18]. Training mothers is one of the effective ways to prevent otitis media [17]. In this regard, educational interventions, campaigns and community-oriented interventions and the necessity of attention of health care providers [20] are suggested in the field of otitis media prevention. Based on documentation, using theories and models to design suitable and successful educational interventions helps us [21]. One of the common and practical models is the PRECEDE proceed model, which was presented in 1970 by Green and Carruthers [22]. The use of this model in educational interventions has been suggested to improve the knowledge, attitude and practice of mothers and empower them in the field of behaviors that promote children’s health.

The current model has been used in the field of controlling iron deficiency in children [23], nutritional support and growth disorder in children aged 6–12 months [24], as well as improving the knowledge and attitude of mothers in the treatment of kids with pneumonia [25]. PRECEDE model classifies the influencing factors on health-related behaviors into three areas including predisposing, enabling and reinforcing factors. Predisposing factors include knowledge, beliefs, values, and attitudes and PRECEDE behavior change and create the necessary motivation for behavior change in a person. Enabling factors include resources, and skills that facilitate the occurrence of a behavior in a person. Reinforcing factors include family, peers, health workers and other people and groups that lead to the continuation of healthy behavior by providing rewards and encouragements [22].

With considering the prevalence of otitis media in children aged 3–18 months and the key role of mothers in preventing middle- ear infections in children, this study was conducted to investigate the effect of the educational intervention by online social media platform on improving mothers’ behaviors towards preventing their children’s otitis media based on the PRECED model.

## Methods

### Study design and participants

This randomized controlled trial study was conducted on 88 (44 in the experimental group and 44 in the control group) mothers of infants covered by comprehensive health service centers of Arak, Iran, from September 2021 to February 2022.

**Fig. 1:**
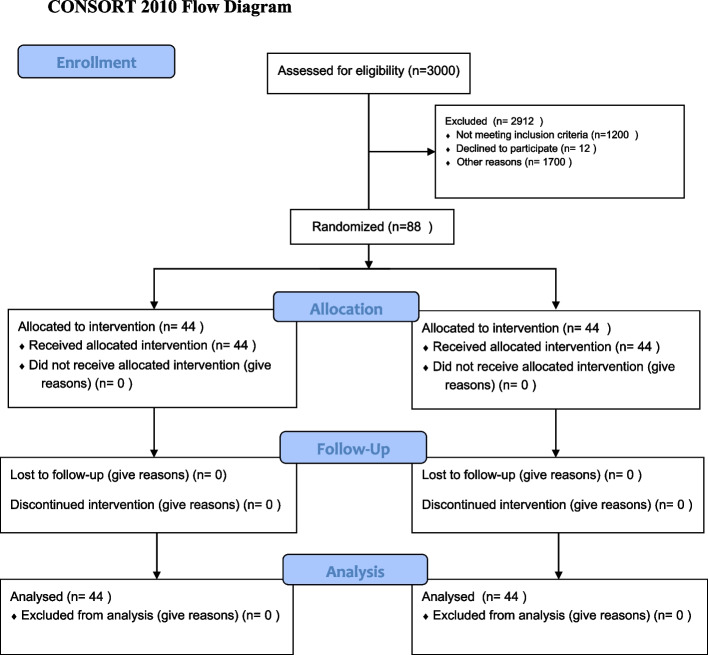
CONSORT flow diagram of the study design. The figure shows the flow diagram of randomized controlled protocol

Stratified random sampling was used for sample selection. According to socio-economic regions, the city was divided into 4 regions. Then 2 health centres were selected by simple random sampling with running a random sample command in SPSS software from each region (totally 8 centers). Furthermore, the participants were selected by systematic random sampling based on electronic registered system in each health center. In each region, based on simple random sampling, control group (*n* = 11) were selected from one center and experimental group (*n* = 11) from another center. Thus, 44 participants were in the experimental group and 44 participants were in the control group (Fig. 1).

### Study setting

This study was conducted on 88 mother–child pairs. They referred to the Health Centers of Arak,Iran for routine preventive child health care (checking child growth, development, and immunization).Arak, the center of Markazi province, is located in center of Iran with a population of around 600,000.In Iran, public health systems for providing primary health cares were established in 1970. Each public health care center was included different health care units (family health, maternity and vaccination, etc.). These primary health care centres provide at least preventive health care (PHC) for all population categories [26].

### Sample size

The sample size considering type 1 error (α = 0.05), type 2 error (ß = 0.2) and average (standard deviation) behavior scores of mothers with children less than 2 years old according to Ahmadi et al.’s study [27] were calculated 4.63 (0.43) before the educational intervention and 4.86 (0.28) about 40 people after the educational intervention. To compensate the loss of sampling, 10% of the determined sample volume (4 samples) was added to the calculated sample volume.

$$n=\frac{\left({s}_{1}^{2}+{s}_{2}^{2}\right){\left({z}_{1-\frac{\alpha }{2}}+{z}_{1-\beta }\right)}^{2}}{{\left(\overline{{x }_{1}}-\overline{{x }_{2}}\right)}^{2}}$$

### Inclusion and exclusion criteria

The inclusion criteria were willingness to participate in the investigation, being skillful at reading and writing, having profile in the health centers, and not suffering from physical, mental, and emotional diseases based on their medical profiles. In addition to having smartphone ownership and ability to communicate on social media also part of the inclusion criteria of the participant in this study. The exclusion criteria were the unwillingness of the participants to continue participating in the study for any reason and who were absent for more than one educational session.

### Data collection tools

The data collection tool was a reliable and valid researcher-made questionnaire [28] that included demographic information (part one), and constructs of PRECEDE model regarding otitis media (part two), and the otitis media preventive behaviors (part three). The core items of the questionnaire were extracted based on several literature review (some literature included Refs. [16, 18–20]). In part two, the constructs of PRECEDE model derived from the available literature and in part. Three, the otitis media preventive behaviors of the mothers derived from the previous similar study [18–20].

#### Part 1- socio demographics characteristics

Variables related to demographic information included mother and child age mother’s educational level (primary school, secondary school, high school, diploma, and academic), mother’s occupation, number of children, child’s gender, self-report economic status (weak, average, good), place of child care, and family history of middle ear infection and child’s exposure to cigarette smoke.

#### Part 2-constructs of PRECEDE model

##### Predisposing factors

Predisposing factors included knowledge and attitude. The knowledge toward otitis media was evaluated through 17 multiple choice items in which every correct answer was given the score 1 and incorrect or “do not know” answer was given a score of 0. Total knowledge score ranged from 0 to 1 and a higher score indicated higher knowledge. Reliability (Cronbach’s alpha) was 0.75.

The attitude toward otitis media was evaluated through included 7 items that were arranged on a 5- point Likert scale (5 = completely agree and 1 = completely disagree). The possible score range was 1–5 and higher score indicate higher attitude. Reliability (Cronbach’s alpha) was 0.70.

##### Enabling factors

The enabling factors included 11 multiple choice items (yes, no and I don’t know) and every correct answer was given one point and wrong answer was given zero point. The possible score range was 0–1 and higher score indicate higher enabling factors. Reliability (Cronbach’s alpha) was 0.73.

##### Reinforcing factors

The reinforcing factors included 6 items that were arranged on a 5- point Likert scale (5 = very much and 1 = very little). The possible score range was 1–5 and higher score indicate higher reinforcing factors. Reliability (Cronbach’s alpha) was 0.81.

#### Part 3- otitis media preventive behaviors

The preventive behaviors were consisted of 11 items on a 5-point scale (4 = always, 3 = most of the time, 2 = sometimes, 1 = rarely, 0 = never). The possible score range was 0–4 and higher score indicate higher otitis media preventive behaviors. Reliability (Cronbach’s alpha) was 0.71.

In addition, the otitis media preventive behaviors mothers have been reported based on the frequency distribution of each behavior in terms of percentage in the scale of always, most of the time, sometimes, rarely and never.

### Measuring tools: validity and reliability

The content validity of the questionnaire was confirmed by expert panel of ten academicians (three general physicians, five health education and health promotion and two health care providers). The mean content validity ratio (CVR) and content validity index (CVI) were calculated to be 0.89 and 0.92, respectively that indicating a good internal consistency.

### Pilot study

The reliability of the questionnaire was assessed by test–retest (2-week interval) in a pilot study among 30 eligible participants and the obtained data were not included in the final analysis. The test–retest reliability coefficient was 0.89 (*P* = 0.001) and each questionnaire completion took approximately 9 min.

In a cross-sectional study (2021) was conducted on 240 mothers of infants referring to health centers of Arak City, Iran, shows 16.2% of mothers had desirable knowledge about otitis media and 67.1% had moderate knowledge. Mothers’ behaviors towards preventing children’s otitis media score was desirable in 58.3% of mothers [28].

### Data collection process

The data were collected from both experimental and control groups before and 3 months after the educational intervention. Questionnaire was made in the google-form using Porsline (Available at URL: https://survey.porsline.ir/s/53Lrr5Y) survey tool. The link of the questionnaire was sent through the WhatsApp social network and was completed as a self-report by participants.

### Intervention

Computer-generated educational intervention based on WhatsApp messenger was carried out. Based on the needs assessment in pilot study [28] before the intervention, the mothers had suggested the WhatsApp messenger to participate in the training classes. Therefore, due to the spread of the Covid-19 disease, the educational intervention and the questionnaires completion were held in WhatsApp. The reason for using the social network WhatsApp; The ability to send all kinds of text, audio and video messages, availability, easy use and no need for special skills in using this social network were for mothers [29, 30].

The experimental group participated in 4 training sessions of 60 min at an interval of 1 week for 1 month (one session per week). In WhatsApp messenger, a group called “Aware Mothers- Healthy Children” was formed for the experimental group. The educational content included voiced PowerPoint, short educational messages, educational photos with short explanations, and short educational videos about middle ear infection that were sent to the mothers of the experimental group. After each training session, group discussion and question and answer were conducted to better understanding of the training contents (Details are shown in Table 1).

**Table 1:** Detailed description of educational sessions for mothers of the experimental group regarding middle ear infection in infants

Meeting	**Learning objectives**	**Learning activity**
First (knowledge)	Providing basic information about the definition of middle-ear infection, the causes of this disease in infants, the symptoms of this infection in infants, and the short-term and long-term complications of this infection (including physical complications and the effects of this disease on the quality of life of the child)	Feedbacks were provided; each mother was encouraged to ask questions. Sending interesting photos with short texts, educational clips with explanations, short messages, voiced PowerPoint, group discussion and question and answer between the trainer and the mothers at the end of the session to strengthen the learning of the mothers
Second (attitude and beliefs about middle ear infection)	Familiarity with the risk factors of middle-ear infection in infants and the role of mothers to prevent the exposure of infants to these factors, how to diagnose and treat middle-ear infection in infants	Feedbacks were provided; each mother was encouraged to ask questions. Sending interesting photos with short texts, educational clips with explanations, short messages, voiced PowerPoint, group discussion and question and answer between the trainer and the mothers at the end of the session to strengthen the learning of the mothers
Third (enabling factors (skills and behavior)	Mother's familiarity with the simple and practical solutions to prevent middle-ear infection in infants in order to strengthen the mother's skills and behaviors in this field	Feedbacks were provided; each mother was encouraged to ask questions. Sending interesting photos with short texts, educational clips with explanations, short messages, voiced PowerPoint, group discussion and question and answer between the trainer and the mothers at the end of the session to strengthen the learning of the mothers
Fourth (enabling factors (access to resources) and reinforcing factors	Mother’s familiarity with reliable sources of information to obtain information about the prevention of middle-ear infection in infants. Mother’s familiarity with the centers that provide free educational and health services in the field of maintaining the health of the ears and hearing of infants. Other family members familiarity with preventive measures for middle-ear infection in infants	Feedbacks were provided; each mother was encouraged to ask questions. Sending interesting photos with short texts, educational clips with explanations, short messages, voiced PowerPoint, group discussion and question and answer between the trainer and the mothers at the end of the session to strengthen the learning of the mothers

Finally, 3 months after the intervention, the post-test was administrated and the data was collected from both groups and was analyzed. With considering that in the process of creating health behaviors in order to change mothers’ attitude and create stability and sustainability in health behaviors, we use the duration of 3 months for participant follow up post-test.

### Data analysis

SPSS version 23 (SPSS, Inc., Chicago, IL, USA) statistical software was used to analyze the data for all tests, the significance level was considered as 0. 05. Descriptive statistics were performed to assess the means and standard deviation (SD), frequencies for categorical variables. Chi-square test were used to compare categorical variables between two groups. Independent samples *t*-test was used to compare the constructs of PRECEDE model and otitis media preventive behaviors score between two groups at baseline and 3 months after the intervention. Paired *t*-test was used to compare the constructs of PRECEDE model and otitis media preventive behaviors score in each group at baseline and 3 months after the intervention.

### Ethical considerations

Written informed consent was obtained from all the participants. Moreover, after the study, the training materials such as the booklets electronic, voiced PowerPoint, short educational messages, and educational photos were given to the control group through WhatsApp social network. The both groups also, received routine child care training through health workers, but the intervention group received the intervention program in addition to the routine training.

The Ethical Committee of Arak University of Medical Science, Iran, approved the study protocol (ID number- IR.ARAKMU.REC.1399.298). The purpose of the study was explained to the participants, and then a written consent was obtained. The explanation and approval of the research delivered online for participation. Moreover social media research interventions require internet quotas to download learning media and researchers in this study by delivering compensated internet quotas try to minimize losses to participants. This study was registered in the Iranian Registry of Clinical Trials (IRCT20210202050228N1). Prospectively registered at 2021-May-21, (2021/05/21) available at: URL: https://en.irct.ir/trial/54073.

## Results

Socio-demographic characteristics of the both groups are presented in Table 2. The mean (SD) age of mothers in the experimental and control group were 28.61 (4.5) and 27.93 (4.4) years, respectively. The mean (SD) age of children in the experimental group was 10.9 (5.8) and in the control group was 10.4 (6.7) months, which did not have a statistically significant difference with each other (*p* value = 0.558).

**Table 2:** Demographic characteristics of participants in control (*n* = 44) and Experimental (*n* = 44) groups

**Variables**	**Category**	**Group**	
		**N (%) Experimental**	**N (%) Control**
Maternal occupation	House wife	34 (77.3)	35 (79.5)
	Employed	10 (22.7)	9 (20.5)
Maternal education	Elementary	2 (4.5)	0 (0)
	High school graduate	18 (40.9)	18 (40.9)
	College education	24 (54.5)	26 (59.1)
Child’s gender	girl	22 (50)	23 (52.3)
	boy	22 (50)	21 (47.7)
Child’ feeding	Mother’s milk	41 (93.2)	37 (84.15)
	Formula milk	3 (6.8)	5 (11.4)
	other	0 (0)	2 (4.5)
Smoke exposure	yes	13 (29.5)	13 (29.5)
	no	31 (70.5)	31 (70.5)
History of otitis media	yes	3 (6.8)	2 (4.5)
	no	41 (93.2)	42 (95.5)

As shown in Table 3, at the baseline, there was no significant difference in the mean scores of constructs of PRECEDE model (knowledge, attitude, enabling factors and reinforcing factors of mothers in preventive behaviors of infants’ middle-ear infection) between two groups (*p* > 0.05). However, after intervention, there was a significant difference in the mean scores of PRECEDE model constructs and behaviors regarding to middle- ear infection between two groups (*p* < 0.001) (Table 3). As shown in Table 4 Comparing of mean and standard deviation of attitude and behavior in the experimental and control group before and after intervention.

**Table 3:** Comparing of mean and standard deviation of PRECEDE model structures in the experimental and control group before and after intervention

**Constructs of PRECEDE model**	**Groups**	**Before**	**After 3 months**	**p Value
		**Mean (S.D)**	**Mean (S.D)**	
Knowledge	Experimental	0.4 ± 0.2	0.8 ± 0.1	< 0.001
	Control	0.52 ± 0.16	0.53 ± 0.14	0.443
	*P value	0.469	< 0.001	
Attitude	Experimental	4 ± 0.6	4.5 ± 0.3	< 0.001
	Control	4 ± .06	3.8 ± 0.5	0.014
	*P value	0.831	0.001	
Enabling factors	Experimental	0.7 ± 0.2	0.8 ± 0.1	< 0.001
	Control	0.77 ± 0.15	0.77 ± 0.14	0.439
	*P* value	0.444	0.007	
Reinforcing factors	Experimental	3.3 ± 0.8	3.9 ± 0.7	< 0.001
	Control	3.5 ± 0.8	3.4 ± 0.7	0.430
	*P* value	0.265	0.006	
Otitis media preventive behaviors	Experimental	3.2 ± 0.5	3.6 ± 0.2	< 0.001
	Control	3.4 ± 0.6	3.4 ± 0.5	0.153
	*P* value	0.007	0.032	

^*^Paired t-test, ** independent samples t-test

**Table 4:** Comparing of mean and standard deviation the difference in scores of attitude and behavior in the experimental and control group before and after intervention

**Constructs of PRECEDE model**	**Groups**	**Before**	**After**	**score difference before and after intervention**	*p Value
		**Mean (S.D)**	**Mean (S.D)**		
Attitude	Experimental	4.0 ± 0.6	4.5 ± 0.31	0.5 ± 0.4	< 0.001
	Control	4 ± 0.6	3.8 ± 0.56	0.1 ± 0.2	
Behavior	Experimental	3.2 ± 0.5	3.6 ± 0.2	0.4 ± 0.5	< 0.001
	Control	3.4 ± 0.6	3.4 ± 0.5	-0.03 ± 0.1	

Due to the significant difference between the mean scores before and after the educational intervention in the control group, separately for those constructs, the difference in scores before and after the educational intervention was calculated for each of the intervention and control groups, so the comparison between them was done with independent t-test

^*^Independent samples t-test

The comparison of frequency distribution based on 4-point scale (always, most of time, sometime, rarely and never) of each behavior to prevent otitis media at the baseline and after intervention between in two groups are shown in Table 5. The results showed a significant increase in the otits media preventive behaviors (such as breastfeeding, using cup instead of pacifiers, avoid exposure to passive smoking, safety and hygiene in child feeding and so on) in the experimental group after the intervention (*p* < 0.001).

**Table 5:** The comparison of frequency distribution of participant’s behavior between in experimental and control group before and 3 months after intervention

**Variables**	**Category**	**Before intervention N (%)**		**After intervention N (%)**	
		**Experimental group**	**Control group**	**Experimental group**	**Control group**
Breastfeeding	Never	2(4.5)	3(6.8)	2(4.5)	4(9.1)
	rarely	1(2.3)	2(4.5)	1(2.3)	1(2.3)
	sometimes	1(2.3)	1(2.3)	0(0)	0(0)
	often	6(13.6)	3(6.8)	1(2.3)	3(6.8)
	always	34(77.3)	35(79.5)	40(90.9)	36(81.8)
	*p Value	0.564		0.020	
Breastfeeding in semi-fowler’s position	Never	2(4.5)	2(4.5)	0(0)	1(2.3)
	rarely	4(9.1)	6(13.6)	1(2.3)	6(13.6)
	sometimes	4(9.1)	3(6.8)	2(4.5)	3(6.8)
	often	16(36.4)	12(27.3)	10(22.7)	7(15.9)
	always	18(40.9)	21(47.7)	31(70.5)	27(61.4)
	*p Value	0.007		0.001	
Removing water from the child ear after showering	Never	2(4.5)	3(6.8)	0(0)	3(6.8)
	rarely	3(6.8)	1(2.3)	0(0)	2(4.5)
	sometimes	5(11.4)	8(18.2)	1(2.3)	8(18.2)
	often	16(36.4)	12(27.3)	14(31.8)	11(25)
	always	18(40.9)	20(45.5)	29(65.9)	20(45.5)
	*p* Value*	0.593		0.001	
Using cup instead of pacifiers	Never	2(4.5)	3(6.8)	0(0)	4(9.1)
	rarely	3(6.8)	3(6.8)	0(0)	1(2.3)
	sometimes	6(13.6)	1(2.3	3(6.8)	3(6.8)
	often	11(25)	6(13.6)	10(22.7)	7(15.9)
	always	22(50)	31(70.5)	31(70.5)	29(65.9)
	*p Value	0.366		0.001	
Avoid exposure to passive smoking	Never	1(2.3)	2(4.5)	0(0)	0(0)
	rarely	3(6.8)	2(4.5)	0(0)	4(9.1)
	sometimes	10(22.7)	4(9.1)	3(6.8)	4(9.1)
	often	12(27.3)	9(20.5)	16(36.4)	13(29.5)
	always	18(40.9)	27(61.4)	25(56.8)	23(52.3)
	*p Value	0.608		0.001	
Safety and hygiene in child feeding	Never	0(0)	0(0)	0(0)	0(0)
	rarely	2(4.5)	2(4.5)	0(0)	1(2.3)
	sometimes	7(15.9)	1(2.3)	4(9.1)	3(6.8)
	often	11(25)	6(13.6)	9(20.5)	11(25)
	always	24(54.5)	35(79.5)	31(70.5)	29(65.9)
	*p Value	0.083		0.033	
Protect child from air pollution	Never	0(0)	0(0)	0(0)	0(0)
	rarely	2(4.5)	0(0)	0(0)	4(9.1)
	sometimes	6(13.6)	2(4.5)	3(6.8)	4(9.1)
	often	15(34.1)	13(29.5)	16(36.4)	13(29.5)
	always	21(47.7)	29(65.9)	25(56.8)	23(52.3)
	*p Value	0.782		0.001	

*N (%)* Number(%)

^*^Statistical test was Chi-square test

## Discussion

To the authors’ knowledge, this study is one of the few online educational interventions based on the PRECEDE model on otitis media preventive behaviors in the children. Few documents have been published about this issue at the time of this study. The results of this study showed that the educational intervention based on PRECEDE model is effective on the knowledge, attitude, behavior, enabling factors, reinforcing factors and mothers preventive behaviors of infants’ middle-ear infection.

The average behavior score of mothers regarding to middle-ear infection in the experimental group increased satisfactorily 3 months after the intervention. This finding is in line with the results of Kashfi et al.’s [31] study, which was conducted based on the PRECEDE model in mothers with children aged 6 to 12 years in the field of preventing developmental disorders. In this study the otitis media preventive behaviors mothers (such as breastfeeding, removing water from the child ear after showering, using cup instead of pacifiers, safety and hygiene in child feeding and so on) it showed that before the training educational in the intervention group, the distribution of the frequency of behaviors has improved from the scale of never and rarely to perform care behaviors to always and most of the time after the training.

After the intervention, the average score of mothers’ knowledge in the experimental group was improved. This improvement was due to the educating mothers about preventative behaviors of otitis media. Meanwhile, there were some text, audio and video messages, and picture for them, and these factors improved the knowledge of the mothers in the intervention group. This finding confirms the results of Saudi et al.’s study [25] and Jalili et al.’s study [23]. In a study by Saudi et al., mobile phone-based education increased the knowledge of mothers about the treatment of children with pneumonia. In the study Bab et al. [32] showed that an increased significant knowledge mother after intervention in control group about oral health in children that this finding not in line with our study and other studies [23, 25]. It should be noted that in the study of Bab et al., the topic of oral and dental health was different from the present study (otitis media preventive behaviors mothers) and the follow-up time of their study was 2 months and shorter than our study.

One of the findings of this study is the increase of the average score of the attitude of the mothers in the experimental group after the educational intervention. This increase can be attributed to the promotion of the mothers’ attitudes about seriousness of otitis media and paying attention to its complications for children and the cost of treatment. In fact, researchers believe that having knowledge alone is not enough to take preventive behaviors. But mothers attitude towards a disease is an important factor in conducting preventive behaviors. In the present study, using group discussions by chat in WhatsApp social network and sharing experiences of mothers and motivating them to prevent the occurrence of otitis media infections in children and introducing the benefits of these behaviors and the complications due to the lack of this care, ultimately increased the attitude of mothers after the intervention. This finding confirms Jalili et al.’s study [23] and Fathizadeh et al. [33]. The attitude of mothers towards behaviors related to the health of the child is one of the predisposing factors for performing the behavior and PRECEDE her behavioral changes.

Finding showed that in the experimental group, the average score of enabling factors increased significantly after the end of the educational intervention. In fact, this enhanced can be attributed to the training method based on PRECEDE model and effective role of virtual education containing educational videos and slides on the skills of performing preventive behaviors against middle-ear infection in infants and instruction how to access services and free information about it. This finding is in line with the results of Kashfi et al.’s study [31], which showed that the enabling factors of mothers in the field of prevention of developmental disorders of children aged 6–12 months was increased. In the present study, presenting an educational program one factors facilitating the behaviors, providing incentives mothers with discussion groups, breaking the behaviors preventive otits media into small steps for facilitate doing, practical education with video educational program, and using the experiences of other mothers with children with otits increased the mothers enabling factors in the intervention group.

Regarding the role of support from family, friends, spouse and health staff in the field of preventive behaviors of otitis media in infants, the average score of reinforcing factors in the experimental group increased from 3.3 to 3.9 after the educational intervention. This finding confirms the results of other studies about preventing febrile convulsion in children [34], oral health in student [35] and breast feeding [36] that was conducted educational intervention based on the PRECEDE model.

In the present study, reinforcing factors such as health staff were influential in increasing the motivation of mothers to follow these individuals. Hence guidance from the health care providers in form of educational classes in health centers for prevent otits media infection in children will be very helpful. Therefore in this study, in order to increase the information of the influential people (family, friends and spouse), some of the materials of the educational booklets electronic, voiced PowerPoint, short educational messages, and educational photos were specifically designed on increasing the information of family and especially the spouse to be effective in adopting the behaviors by mothers. Finally raising knowledge as well as other constructs of PRECED model, including attitudes, enabling factors and reinforcing factors all led to increased skills and preventive behaviors by mothers.

One of the implications of the present study for health planners and policy maker in health promotion is to produce standard educational content based on the needs assessment with theory center and use them by health planners with the aim of providing training by health personnel in health centers and doctors and nurses in pediatric in clinics.

## Strengths and limitations

One of the strengths of this study was the use of various teaching methods and the combination of face-to-face (in the form of a video call) and virtual training, as well as the random selection of samples. Moreover the use of PRECEDE model as a community‑based model for design, implementation, and evaluation of intervention and identification of knowledge, attitude, predisposing, enabling, and reinforcement factors about preventive behavior otits media in children is one of the strengths of the present study. In this study focus on children under two years as a high risk group for otit media, so paying attention to this target group is also one of the strengths of the research.

There were some limitations to this study. Data collection was self-reported that could be subjected to recall and social desirability biases. This limitation was resolved by allocating sufficient time and explicit expression of the objectives of study, and gathering information along with interviewing. Also, we followed up the mothers for 3 months as the longer follow up may lead to more accurate outcomes. Therefore, it is recommended that the educational program and the follow-up of mothers be continued for a longer period of time and the outcomes be evaluated in longer periods after the intervention.

## Conclusion

According to the results of the present study, the educational intervention based on the PRECEDE model can improve knowledge, attitude, enabling factors, reinforcing factors and ultimately improve the mothers’ otitis media preventive behaviors. The use of theory-based educational interventions as well as the use of virtual space seems beneficial to educate mothers in the field of behaviors related to children’s health. Therefore, it can be said that it is necessary for health service providers in primary care centers to consider practical and beneficial educational programs about otitis media prevention in children.

## Data Availability

The datasets analyzed during the current study available from the corresponding author on reasonable request.

## Abbreviations

CVR: Content Validity Ratio
CVI: Content Validity Index
